# Trends and gaps in biodiversity and ecosystem services research: A text mining approach

**DOI:** 10.1007/s13280-022-01776-2

**Published:** 2022-09-03

**Authors:** Viktoria Takacs, C. David O’Brien

**Affiliations:** 1 Department of Zoology, Faculty of Veterinarian and Animal Sciences, Poznan University of Life Sciences, Wojska Polskiego 71/c, 60625 Poznan, Poland; 2 NatureScot (Scottish Natural Heritage), Inverness, IV3 8NW UK; 3 Royal Botanic Garden Edinburgh, Edinburgh, EH3 5LR UK

**Keywords:** Biological diversity, Nature’s contribution to people, Research policy interface, Research trends, Research weaving, Topic modelling

## Abstract

Understanding the relationship between biodiversity conservation and ecosystem services concepts is essential for evidence-based policy development. We used text mining augmented by topic modelling to analyse abstracts of 15 310 peer-reviewed papers (from 2000 to 2020). We identified nine major topics; “Research & Policy”, “Urban and Spatial Planning”, “Economics & Conservation”, “Diversity & Plants”, “Species & Climate change”, “Agriculture”, “Conservation and Distribution”, “Carbon & Soil & Forestry”, “Hydro-& Microbiology”. The topic “Research & Policy” performed highly, considering number of publications and citation rate, while in the case of other topics, the “best” performances varied, depending on the indicator applied. Topics with human, policy or economic dimensions had higher performances than the ones with ‘pure’ biodiversity and science. Agriculture dominated over forestry and fishery sectors, while some elements of biodiversity and ecosystem services were under-represented. Text mining is a powerful tool to identify relations between research supply and policy demand.

**Supplementary Information:**

The online version contains supplementary material available at 10.1007/s13280-022-01776-2.

## Introduction

The adverse impacts of human activity on nature are being increasingly recognised, with some authors referring to a biodiversity crisis (Western 1992; Eldredge 2000; IPBES 2019). There has also been a greater understanding of the importance of biodiversity for people as an asset that should be protected and maintained for our needs and those of future generations. This linkage has been acknowledged in both scientific publications and in policy at national and international levels. The term ‘biodiversity’ first attained prominence in the international policy agenda at the Earth Summit (CBD 1992), which listed three components: species, ecosystems and genetic diversity. The vital role of biodiversity for humanity was further expounded in the Millennium Ecosystem Services report (MEA 2005), which has led to a growth in research in the ecosystem services (ES) that biodiversity provides.

The Convention on Biological Diversity (CBD 1992) is a major driver of policy with 196 signatories. Twenty targets were set when the parties met at Nagoya, Aichi prefecture, Japan in 2010 and ecosystem services feature prominently in the Strategic Plan for Biodiversity (CBD 2010; United Nations 2011). However, few countries have been able to meet even half of the targets and at a global level, none were fully met (CBD 2020). Other assessments such as the Intergovernmental science-policy Platform on Biodiversity and Ecosystem Services (IPBES 2019) and The Economics of Ecosystems and Biodiversity (TEEB) (Sukhdev et al. 2014) also link the two concepts and have come to similar conclusions regarding the ineffectiveness of actions to date. In response, the CBD parties are working to produce new targets that will track progress to 2030 and beyond (CBD 2021a). A key point in early discussions has been understanding the benefits humanity gains from ecosystem services (CBD 2021b). While these policy documents highlight a need for evidence, the link between evidence needs and recent publications is not clear. Better understanding of what research has been carried out, and what opportunities and gaps exist will help funding agencies to direct resources where they can have the greatest impact for biodiversity and the benefits it provides.

Biodiversity conservation and the concept of ecosystem services are not always complementary. Thus, enhancing ecosystem service provision does not necessarily lead to biodiversity conservation or sustainable resource management (Oguh et al. 2021). In general, there are three ways of treating biodiversity within the ecosystems services framework (Mace et al. 2012); (i) biodiversity and ecosystems services are treated as synonyms; (ii) the “conservationist perspective” includes biodiversity as an ecosystem service by itself; or (iii) biodiversity can sometimes be a final ecosystem service—e.g. wild relatives of cultivated crops, which can be a source of improvement for domesticated species, or medicines from wild plants (Mace et al. 2012).

During the evolution of the ecosystem services concept, the role of biodiversity in the ecosystem service categories has changed. At the beginning of ES theory summarised in the MEA (MEA 2005 highlighted earlier by Constanza et al. in 1997), biodiversity was mentioned as a category of ES (within the category of “supporting” services). Later, “The Economics of Ecosystems and Biodiversity” (TEEB) assessment changed the name of the category to “habitat and supporting services”. In an effort to avoid redundancy and counting supporting services (such as biodiversity itself) twice, the hierarchical scheme of ecosystem services devised by the Common International Classification of Ecosystem Services (CICES 5.1.) (Haines-Young and Potschin 2018) does not treat biodiversity as a separate Ecosystem Service itself and does not mention it directly among the ES categories. The category most directly related to biodiversity is within Cultural ES, category 3.2.2.1 “Characteristics of living systems that has an existence value”. In addition, biodiversity is included in 2.2.2.3. “Maintaining nursery populations and habitats (Including gene pool protection)”. However, it has been argued that nature’s contribution to people, and thus ES, cannot be understood without considering biodiversity (Maes et al. 2016).

The need to base policy on sound evidence and the need for robust indicators to underpin this evidence is becoming increasingly recognised by policy-makers (e.g. Wentworth and Henly 2021). This is particularly important as nations negotiate the framework for global biodiversity (CBD 2021a, b). There are additional links that should be explored in the field of biodiversity indicators of sustainability (Hillebrand et al. 2018) and biodiversity’s links to ecosystem functioning (Balvanera et al. 2006, 2012; IPBES 2018). As a result of the urgent need for scientific evidence on nature’s contribution to people as well as on the state and trends of biodiversity, the quantity of scientific publication in these fields has increased rapidly (McDonough et al 2017; IPBES 2018, 2019). This process is reflected in the increased frequency of these words in policy documents as well as in peer-reviewed publications (McDonough et al. 2017; Czucz et al. 2018, 2021). As a result of the extremely large amount of material, it is not practical to perform a “traditional” systematic review in order to identify trends, and ‘hot and cold’ topics. We propose a method that is novel for this scientific field: text mining with a topic modelling approach.

Bibliometric analyses have been incorporated into reviews (research weaving) as supporting material to systematic reviews (Nakagawa et al. 2019). Text mining techniques have gained popularity in summarizing trends and giving guidelines in fields where published information is too great to review all publications. Text mining as a technique has become particularly popular in fields where a rapid increase in published material has occurred, such as information technology and computing, mathematical sciences, linguistics, medical and educational sciences (Nakagawa et al. 2019; Westgate et al. 2020). However, in the fields of biology, ecology and behavioural sciences, there are relatively few publications based on this technique (Jung and Lee 2020).

The aim of this paper is to provide a comprehensive analysis of how biodiversity and ecosystem services have been considered together in peer-reviewed papers from 2000 to 2020. By doing so, we seek to provide evidence of interest and, potentially, gaps in research. We believe this will be of value in the development of research to support policy in these two key topics. Our research question is: which are the most frequent research fields, and how have they evolved in time. This is the first time that the topic modelling technique has been applied for ecosystem services and biodiversity, allowing us a first comprehensive analysis of the development of scientific interest on these topics.

## Materials and methods

In our article’s search and research question formulation, we partially followed the ROSES protocol (RepOrting standards for Systematic Evidence Syntheses; Collaboration for Environmental Evidence 2013) as a basic guideline for systematic reviews and systematic mapping). The protocol contains “research search”, “article screening and appraisal”, “critical appraisal”, “data extraction” and “data synthesis and presentation”. We followed the ROSES protocol for systematic mapping concerning literature search; however, we diverged from the protocol in the phases of “article screening and study appraisal” as no further screening strategy, or reduction of the basic collection of the articles was applied. Our method does not allow for data extraction, while presentation and synthesis of the data also contained elements of the protocol, “narrative synthesis” and identifying “knowledge gaps”.

Using Web of Science (WOS) on the 9th of March 2021, we simultaneously collected all entries where abstract, article title or keywords contained (ecosystem AND service*) AND [biodiversity OR (biological AND diversity)]. Our analysis focussed on peer-reviewed original research papers and reviews, published in the English language. Book chapters, book reviews, conference materials, as well as grey literature, were excluded from the analyses. We restricted our analyses to the period 2000–2020. Altogether, 15 310 publications were included in the corpus of our analyses.

All analyses were performed in R 3.0.3. (R core team 2021). First, we exported all articles found in WOS into a single table (a maximum of 500 articles can be exported at once so we combined several tables into one). We then removed duplicated documents that were present in the WOS dataset. Abstracts were converted into a “tidy” format; a table with one token per row (Silge and Robinson 2016: a token is a meaningful unit of text, such as a word, that we are interested in using for analysis). To achieve this, we first converted the dataset with the help of packages dplyr (Wickham et al. 2020) and tidytext (Silge and Robinson 2016). After this, we cleansed the dataset for common words such as articles, “the” “of” ‘a”, so that only the meaningful words remained (stopwords function in tm package Feinerer et al. 2008). Additionally, we removed the keywords for which the literature search was conducted and commonly added tags (e.g. Elsevier Rights Reserved) via filtering the word matrix.

We obtained the main research topics with the help of topic modelling. This technique is equivalent to clustering in text analyses (reviewed by Westgate et al. 2015). Topic modelling reduces a corpus of scientific documents to a set of topics, and makes it possible to compare them and analyse temporal changes of these topics as a means of gaining insight into the development of scientific fields (Griffiths and Steyvers 2004). Topic modelling was conducted via Latent Dirichlet Allocation (LDA) with the help of R package “topicmodels” (Grün and Hornik 2011). LDA is a machine learning based method for allocating the documents to “topics” (Blei et al. 2003; Siege and Robinson 2017). “Topics” are mixtures of words that occur together in one document with higher probability than they do with others. Each document is a mixture of topics, and a single word might belong to several topics. As a result, LDA is a sort of “fuzzy clustering”. LDA finds the group of words that are associated with each topic and also determines the mixture of topics that describe a document. As a result, we obtain the probability of a document belonging to each of k topics (*γ* or prevalence) and the probability of each word belonging to a topic (*β*) (Murakami et al. 2017; Perry and McGlone 2021). Thus, LDA is a mathematical method for finding the mixture of words that is associated with each topic, while also determining the mixture of topics that describes each document (Silge and Robinson 2017).

The number of clusters (topics) is typically pre-defined by the user as based on an ‘optimal’ number of topics for a given set, and is unambiguous (Silge and Robinson 2017). In order to define this optimal number we ran the programme with several pre-defined initial numbers of topics (*k* = 5, 9, 10, 20, 30) and we chose the largest number of categories that grouped articles such that they belonged nearly exclusively (maximal level of gamma over 0.999) to one topic. In this way, we obtained nine main topics, which gave us meaningful segregation of the published material. For all publications, we assigned the topic that best fitted (highest gamma scores). This allowed us to test the temporal patterns in publication number and citations.

Our analyses were based on abstracts only as it was not practical to obtain the full texts for over 15 000 articles. To bias check the assignment of documents based on abstracts, we downloaded full texts of the 20 best fitting articles for each topic (180 articles). We completed topic modelling on these full texts, and compared the division of articles with the results to the analyses based on the abstracts. After topic assignment (each article to the topic), we were able to observe temporal changes in publication numbers, topic specifics (average gamma values), citation metrics, and temporal changes. The significances of temporal trends, and relations between the amounts of citations and articles were tested by linear regression models (annual mean values of number of articles (gamma), number of citations as a function of time, and annual mean number of citation as a function of annual mean number of articles). All these metrics served for comparing topics and identifying the “hot” fields as well as temporal changes. “Hot topics” are of two types: those topics that occur most frequently and those that appear in the articles that have the highest number of citations. “Cold topics” have the opposite characteristics, appearing infrequently or being cited less often than the other topics considered.

Finally, to be able to achieve a narrative synthesis of the topics, we applied a novel approach (a quantitative tool for comparing the content of the nine topics). We inspected the occurrences of certain pre-defined fields (of interest to us) within the most frequent word matrices in the nine topics. We searched terms among the first hundred most frequent words (according to beta values) within the nine topics. Our chosen fields of interest were as follows: economically important sectors (agriculture, forestry and fishery), policy, nature conservation, economics, taxonomic representation and ecosystem service types (using CICES categories). We compared the sum of beta values (probabilities of belonging to the given topic) of words belonging to those pre-defined fields. This approach gave us further insight into important fields as well as allowing us to identify some knowledge gaps.

**Fig. 1:**
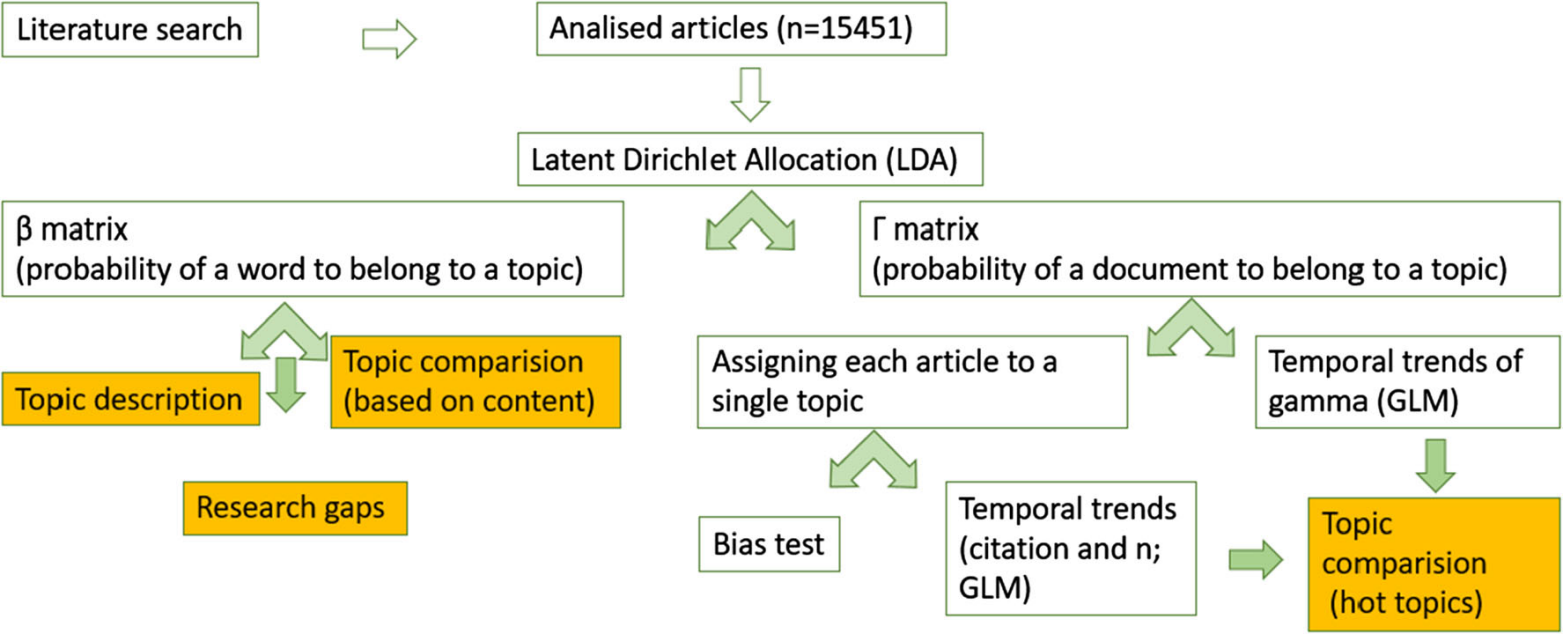
A summary of applied methods and obtained information

A summary of the methods applied is presented on Fig. 1.

## Results

Our search found 15 451 peer-reviewed scientific articles that contain the terms “biodiversity” and “ecosystem services”. Of these articles, 141 did not have abstracts that were available in the WOS database: as a result, we analysed 15 310 documents. These documents contained 47 754 distinct terms, which served as a base of the topic modelling.

**Fig. 2:**
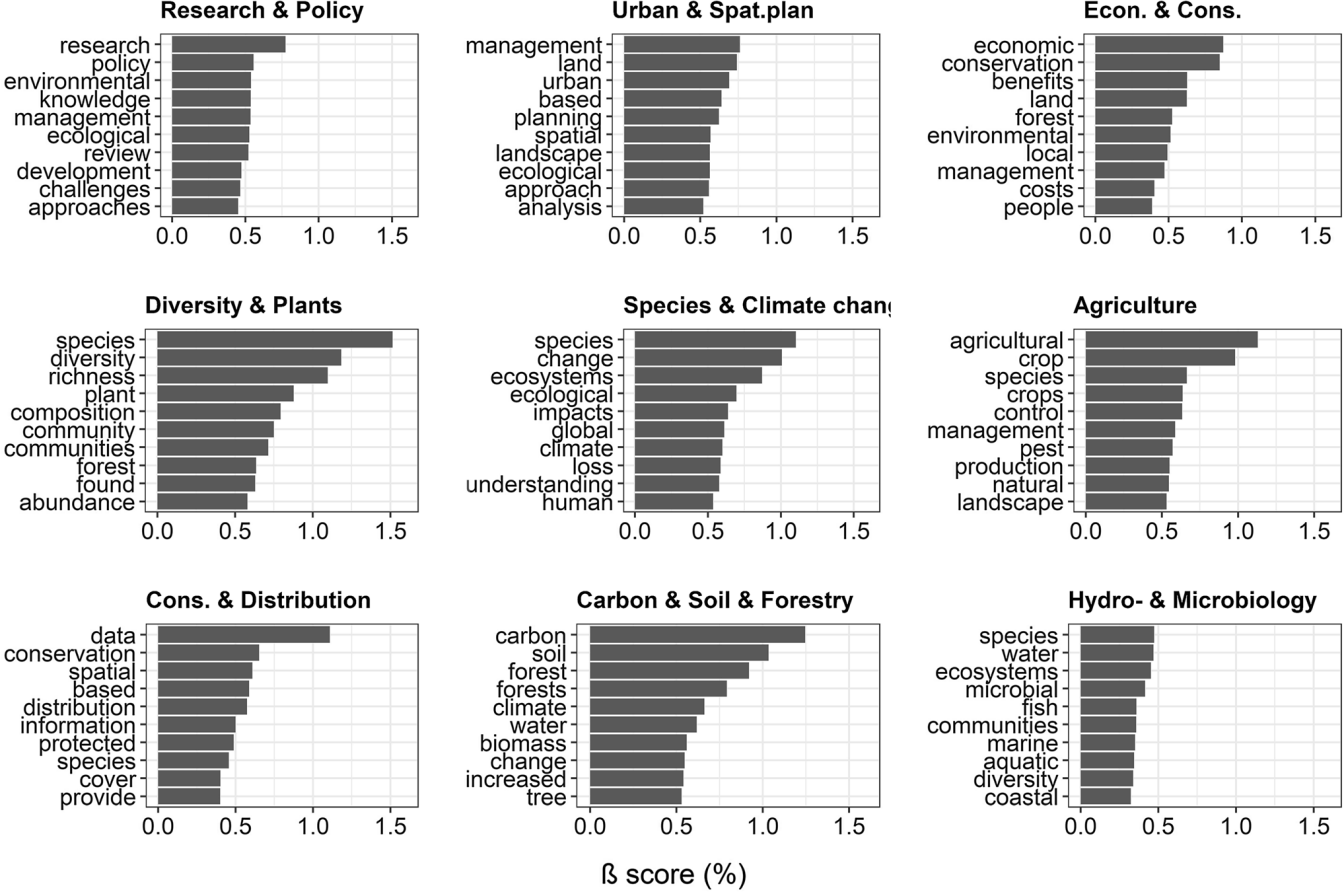
Top ten most frequently occurring words in the nine main topics (the highest *β* values from the topic modelling,). All topics are defined by the matrix of frequencies of word occurrences. Each document can be characterised by its probabilities of belonging to certain topics. Consequently, topics are not exclusive, rather they are matrices of co-occurring words

Topic modelling revealed nine distinct topics. The top 10 most frequent words from each topics are presented in Fig. 2. [for the top 100 most commonly occurring terms, see Supplementary Material (S1)]. The full texts of the twenty best fitting articles from each topic were analysed, applying the same methods. We compared clusters and article divisions in both abstract and full-text analyses. Out of the “top 20 best fitting full texts” (174 full-text documents as six of the “top bests” were not available), only 10 were not assigned into the same group as the abstract had been. This means that topic modelling based on abstracts gave a 94% identical assignment (in the case of the best fitting articles).

Topics are ordered according to the number of articles, after assigning the articles to the topic to which it has the highest probability of belonging, based on the article abstract word matrix (Table 1).

**Table 1:** Description of topics. The topics were the results of LDA analyses. Ranges of *β* % values were calculated on the basis of the top 10 most frequent words in each topic. Mean numbers of articles belonging to each topic (*n*), mean topic prevalence (*γ*) with standard error (SE) and mean numbers of citations per article within topics were calculated after assigning each article to the topic with highest gamma scores

Topic name	Γ (SE)	*n*	Citations (SE)	*β* % range
Research & Policy	0.615 (0.004)	2596	41.134 (2.04)	0.5–0.7
Urban & Spat. Plan	0.546 (0.004)	2141	29.075 (1.42)	0.5–0.7
Econ. & Cons	0.547 (0.005)	1935	35.013 (4.16)	0.3–0.9
Diversity & Plants	0.548 (0.004)	1745	19.605 (1.09)	0.6–1.5
Species & Climate change	0.515 (0.004)	1711	80.520 (5.82)	0.5–1.1
Agriculture	0.622 (0.003)	1450	36.988 (2.16)	0.5–1.2
Cons. & Distribution	0.530 (0.004)	1306	24.890 (2.75)	0.3–1.1
Carbon & Soil & Forestry	0.536 (0.005)	1282	28.423 (2.01)	0.6–1.2
Hydro- & Microbiology	0.567 (0.005)	1144	28.563 (2.0)	0.2–0.5

In case of three topics (“Research & Policy”, “Urban & Spat. Plan”, “Hydro- & Microbiology”), characteristic words (*β* values) are evenly distributed. For the remaining topics (“Diversity & Plants”, “Species & Climate change”, “Agriculture”, “Carbon & Soil & Forestry”), the group is characterised by a dominance of the titular words (Fig. 2).

Overall number of publications in all topics increased after the Millennium Ecosystem Assessment in 2005 (annual numbers 2000–2021 are presented as a function of number of published articles).

**Fig. 3:**
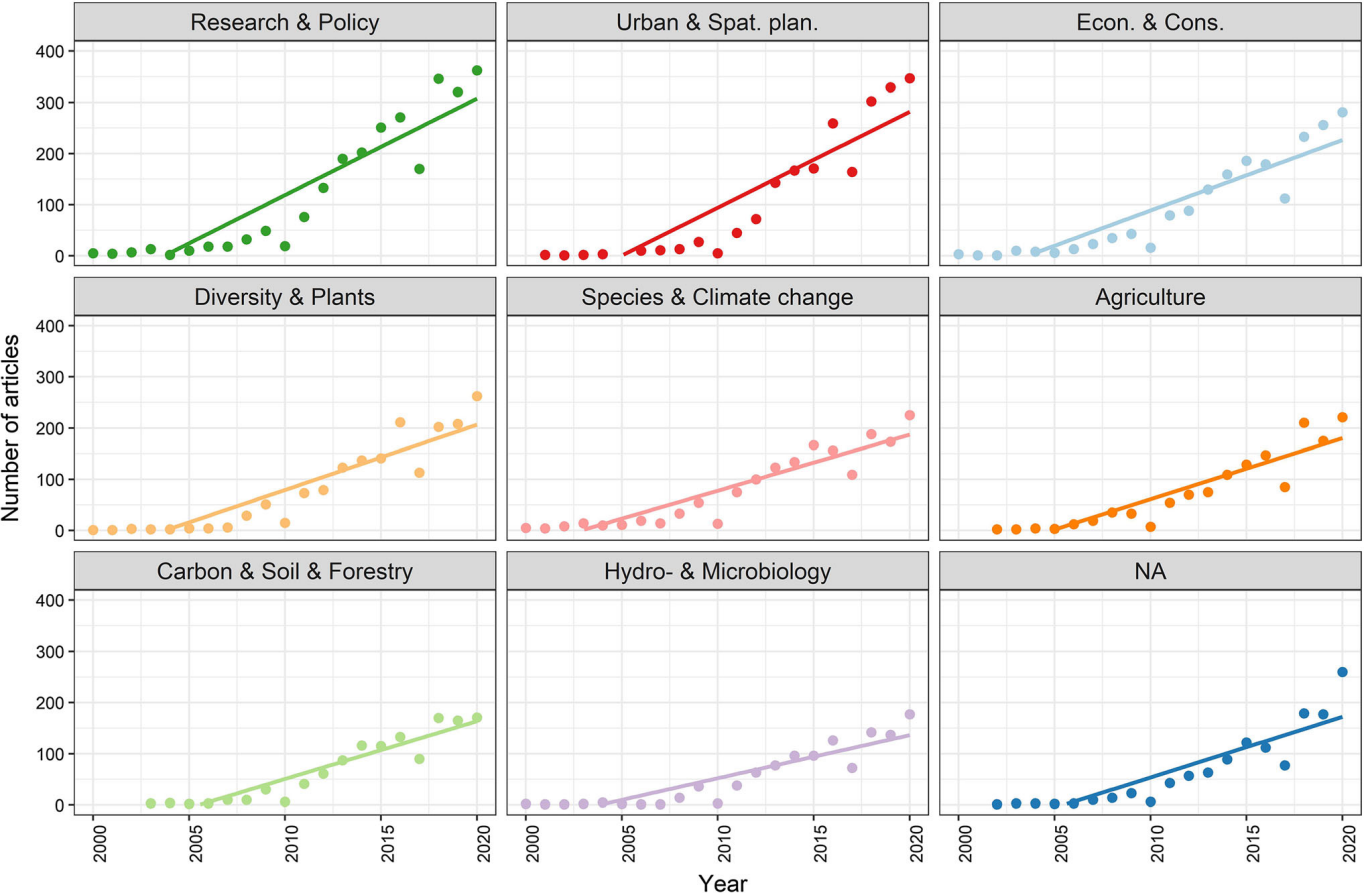
Temporal changes in the number of articles in the nine topics. Points show the annual sum of articles. Regression lines are drawn in cases with significance at *p* < 0.05 level

There was an increase of publications in every topic (Fig. 3); however, this was most rapid in the case of topics” Research & Policy” and “Urban & Spat. plan.”.

**Fig. 4:**
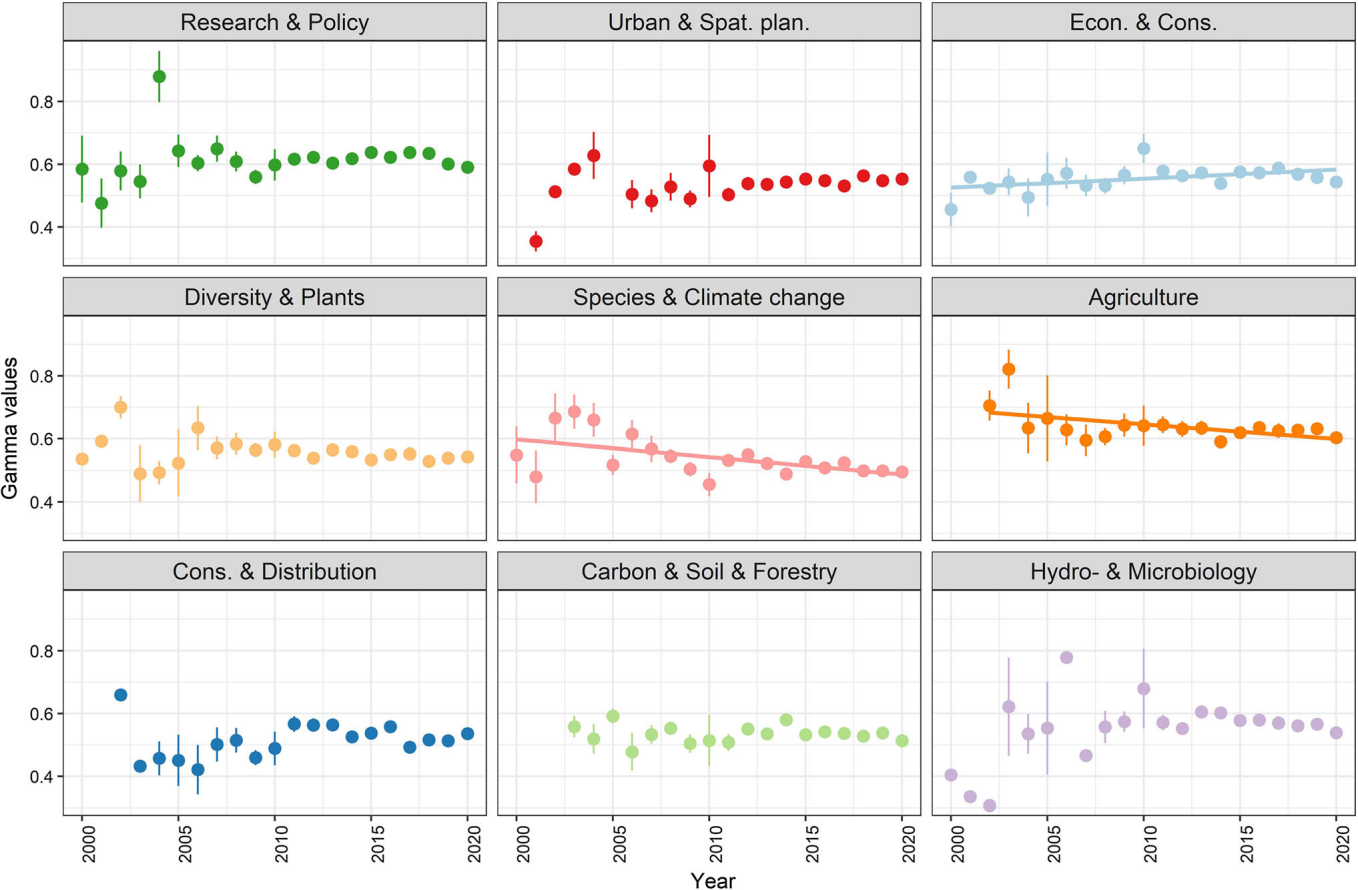
Changing topic prevalence over time. Each article was assigned to the topic with the highest gamma (prevalence) value. Gamma value shows how “well” the articles fit into the given topic. Points show the annual mean gamma values, with point ranges showing standard errors. Regression lines are drawn in cases with significance at *p* < 0.05 level

The annual changes of topic prevalence (Fig. 4) show a significant decrease in the case of the “Agriculture” and “Species & Climate change” topics and a slight increase in the case of “Economics and conservation”. *F* and *p* values are presented in Supplementary Material S2.

The annual total of citation scores for articles in most topics show a nonlinear pattern in time, with a peak around 2015. This peak may in part reflect a lag before the most recent articles are cited.

**Fig. 5:**
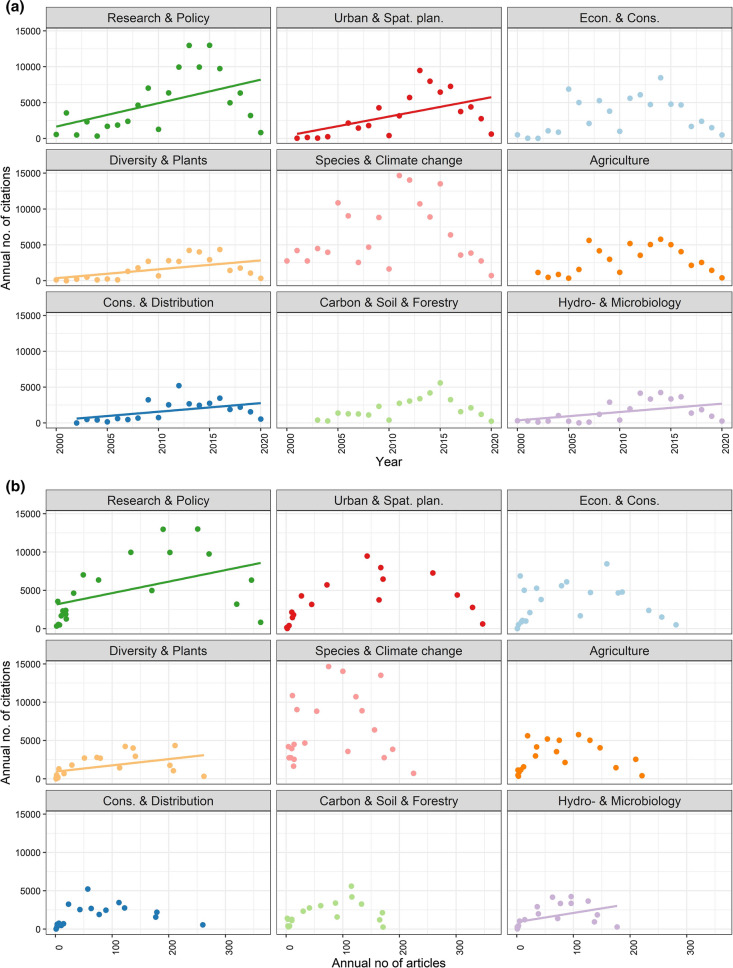
**a** Changing number of citations over time. The points indicate the annual sum of citations and number of articles in a given topic. Regression lines are drawn in cases of significant trend at *p* < 0.05 level. **b** Changing number of citations as a function of article numbers (**b**). The points indicate the annual sum of citations and number of articles in a given topic. Regression lines are drawn in cases of significant trend at *p* < 0.05 level

The number of citations significantly increases in time in the case of five topics, (at *p* < 0.05 level) (presented in Fig. 5a): “Cons. & Distribution”, “Diversity & Plants”, “Hydro- & Microbiology”, “Research & Policy” and “Urban & Spat. Plan.”. Of these topics, in the case of first three, there was an increase in citations reflected in an increase in articles (Fig. 5b); “Diversity & Plants”, “Research & Policy” and “Hydro- & Microbiology” *F* and *p* values are presented in Supplementary Material S2. This suggests that in the case of “Cons. & Distribution”, the increasing trend in citations is probably due to a small number of highly influential articles.

The highest number of articles belongs to the topic “Research & Policy” followed by “Urban & Spat. Plan” and “Economics and conservation” (see also Table 1) with the first two topics showing the highest increase rate over time. Average topic prevalence (gamma) is the highest in “Agriculture” and “Research and Policy” topics, and lowest in “Species & Climate change” and “Carbon & Soil & Forestry”, though both “Agriculture” and “Species & Climate change” show a decrease over time. As by definition, gamma is the probability of words belonging to the topic, a decreasing gamma value means a decreasing trend in topic specificity.

The highest overall number of citations per publication was shown by articles from the group “Species & Climate change” (*n* = 80.5), followed by “Research & Policy” (*n* = 41.1) and “Agriculture” (*n* = 37.0). In the cases of topics “Species & Climate change” and “Agriculture”, high citation numbers are not in conjunction with temporal changes or with article numbers, suggesting that there are few very influential articles in these topics with many citations.

All the metrics we investigated might contribute to identifying “hot topics”. The topic which performs best or close to best in most metrics is “Research and Policy”. In all other cases, however, different metrics favour different topics. This suggests that for these other cases, there may be a mismatch between what topics are preferred by authors at a given time (i.e. number of articles) compared with those which have the most influence, as recognised by number of citations.

Within the top 100 words from each topic (Supplementary material S1), we checked the frequencies of certain terms related to chosen topics; these frequencies are presented in Table 2.

**Table 2:** Chosen thematic groups of terms among the first 100 most frequent words within the nine topics. Sum *β* (%) is the summarised *β* % from topic modelling (denoting the probability of appearance of the given term in the article abstract that belongs to this topic)

Group of terms	Terms within the 100 characteristic words for the topics	Sum *β* (%)	Topics with the given groups of terms (*β* %)
Agriculture	Agricult*, Crop* farm* yield*	6.663	“Agriculture” (5.610) “Econ and Cons”(1.053) “Research & Policy” (0.239)
Forestry	Tree*, timber, wood, deforestation, forest	5.717	“Carbon & Soil & Forestry” (3.090) “Econ and Cons.” (1.928) “Diversity & Plants” (1.190)
Fishery	Fish, aquatic, water*, freshwater, sea, wetland*	3.758	“Hydro- & Microbiology” (1.864) “Econ and Cons.” (0.206) “Cons. & distribution” (0.542) “Carbon & Soil & Forestry” (0.617) “Urban & Spat. plan.” (0.368)
Policy	Policy, implementation, stakeholders, sustainable, approach, challenges	6.409	“Research & Policy” (3.470) “Econ and Cons.” (1.229) “Urban & Spat. plan.” (1.443) “Cons. & distribution” (0.221)
Economics	Income, cost*, benefit*, pay*, PES, financial, evaluate, incentives, market	3.569	“Econ and Cons.” (2.441) “Science & Policy” (0.181) “Urban & Spat. plan.” (0.947)
Nature conservation	Natur*, conservation, protect*, threatened	8.060	“Econ. and Cons.” (2.087) “Cons. & distribution” (2.032) “Research & Policy” (1.033) “Urban & Spat. plan” (1.191) “Diversity & Plants” (0.562) “Agriculture” (0.842)
Taxonomic categories of biota	Bee*, flower, insect*, bird, reef, coral, invertebrates, bacterial, microbial	4.011	“Hydro- & Microbiology”(2.213) “Agriculture” (1.798)
CICES ES categories	Climate, invasive, cultural, production, soil, pest control, pollinat*, organic carbon, sequestration, enemies, nutrient flow, biomass, erosion, timber, emission*, reduction, invasive	12.428	“Carbon & Soil & Forestry” (5.556) “Agriculture”(3.659) “Species & Climate change” (0.802) “Hydro- & Microbiology” (0.728) “Urban & Spat. plan.” (0.664) “Econ. & cons.” (0.515) “Diversity & Plants” (0.294) “Research & Policy” (0.207)

Agriculture and forestry-related terms appear with a relatively high frequency (Sum β%) compared to fishery sectors.

A high frequency was attributed to terms related to nature conservation and species protection. These words were among the most frequent 100 terms in the case of six topics, with the highest frequently in “Econ. & Cons.” and “Cons. & Distribution”, and to a smaller extent in topics “Research & Policy”, “Urban & Spat. plan.” and with low frequency in “Diversity & Plants” and “Agriculture”.

“Policy” and related terms were characterised by a high overall frequency. These words were frequent mainly in topics “Science & Policy”, “Econ. & Cons.”, “Urban & Spat. plan” and to a lesser extent “Cons & Distribution”.

Terms related to Economics appear somewhat less frequently overall: mainly in topics “Econ. and Cons.” and to a lesser extent in two other topics “Urban and Spat. plan” and “Research & Policy”.

Ecosystem service categories appeared (based on the terminology of CICES) with a relatively high frequency (Sum *β* = 12.463%). However, in this case, we have certain limitations as our categorisation is based on single words, while ES categories are more complex. On the other hand, parts of these ES terms might be in other contexts. As a result, in our matrix, certain terms might be an over-represented—e.g. soil organic carbon might appear with 3 different beta values, and some other terms might be not detected in the word matrix. Nevertheless, the appearance of these terms in certain topics is indicative of their prevalence, particularly in contrast to the low frequency of certain services (wind shelter, nursery populations).

Taxonomic names of biota appeared in two topics only: “Hydro- & Microbiology” and “Agriculture”, with summarised *β* value of 4.011%. Only birds, fish, insects, invertebrates, bacteria and microbiota and flowers were mentioned.

## Discussion

Over the past 20 years, the increasing number of publications in the fields of ecosystem services and biodiversity makes it impossible to summarise them using the traditional methods of meta-analyses or systematic reviews. The presented method, which allows analysis of trends and temporal patterns related to certain topics, is proposed for overcoming this problem. In our analysis, we have contributed to knowledge on the relationship between ecosystem services and biodiversity research, showing publication trends and hot topics within this field. This is of particular importance when we consider the need to match research with wider societal needs and pressure for policy to be evidence-based (Sutherland et al. 2004). We discuss the advantages and limitations of the applied method, and following this, we compare and suggest interpretations of the topics’ performances.

While topic modelling has already proved to be of value in other areas of science and technology, its potential is yet to be exploited in ecology (Jung and Lee 2020). We believe our paper shows it can be a useful tool to help understand trends, highlight gaps in research and thereby better align research with policy priorities. It is able to handle large numbers of articles: in this case over 15 000. Our analyses were based on abstracts and included all documents containing the search terms. A traditional review or text mining of full articles could be more accurate; however, in the case of more than ten thousand documents, the review process necessarily would end up with a subjective choice in order to decrease the number of articles at some point. Abstracts can be easily downloaded and analysed in large numbers as well, allowing us to analyse a large number of documents at the same time. There was only a 6% difference in classification result between abstracts and full texts, therefore, we consider abstracts as good representative material for reviews. However, we should keep in mind that our bias test is based on analyses of the most characteristic articles from each topic; it may well be that other sets of full-text articles would not give such a good fit. We propose that any future analyses should also consider the differences between topic model results based on abstracts and full-text documents. A further area of investigation might be grey literature (including technical reports and project findings), which may be a better reflection of how practitioners are responding to research and be a means of understanding the “research—implementation gap” (Cadotte et al. 2020. This analysis might be aided by repositories such as Applied Ecology Resources (Cadotte et al. 2020). At the same time, we note that there will be challenges to analysing documents that may not have summaries or abstracts, or that may contain duplicates of work published elsewhere. Generally, Topic modelling has many advantages in reviewing published materials on given topics; however, there is a difficulty in interpretation of such wide material. We proposed comparing representation of certain fields in the topics based on the frequency of appearance as a simple way to objectively summarise the results.

In our case, topic modelling of abstracts suggested a minimum of nine distinct “topics” at the interface of biodiversity and ecosystems services research. At the moment, there is no standardised method for choosing the numbers of clusters. In this study, the number of the topics was the largest number of clusters where at least one article showed above 99% fit; this is slightly different from other authors (e.g. the approach of Westgate et al. 2015, 2020), where a higher number of clusters was chosen. We recognise that there may be advantages to a standard method for choosing the number of topics in topic modelling but also note that authors may prefer to set a number of topics based on the planned uses of their analyses.

The number of publications in all topics showed a strong increase after the publication of the Millennium Ecosystem Assessment (2005; e.g. “species and climate change” and “agriculture”), and in the CBD Aichi conference in 2010 (e.g. “research and policy” and “cons. and distribution”), which suggests an effort by researchers to make papers that are policy-relevant. This impression is further supported by the finding that “Research & Policy” was generally the “hottest” topic in terms of applied metrics (number of publications, number of citations and a constant, relatively high level of topic prevalence). It will be instructive to see if there are similar upturns or changes in “hot topics” following the Kunming CBD conference in 2022. Policy and related terms appeared among the most frequent words in the case of three other topics, two of which also showed a high performance based on the applied metrics. These results reflect recognition by the research community of the increasing need for considering biodiversity and ecosystem services in policymaking development and practice, as stated in several recent documents (IPBES 2016, 2018, 2019; CBD 2021b). Outside of the topic “Research & Policy”, it was not so straightforward to assign “hot topics” as different metrics gave differing results.

The increasing tendency to include economic and human dimensions into ecosystem services and biodiversity conservation issues has been reflected in recent policies such as CBD (CBD 2021b), EU biodiversity strategy 2030 (EC 2020) or the Green Deal (EC 2019). These policies demonstrate a shift from nature conservation aiming at nature’s intrinsic value (stated in EU habitat directives as an example) towards a “natural capital” approach (Buijs et al. 2022; Hermoso et al. 2022). This shift in perception led from a protective conservation strategy towards treating nature as an asset—a practical development of the ecosystem services approach (Hermoso et al. 2022). The fact that the first three most published topics “Research & Policy”, “Urban & Spat. plan.” and “Economics & Cons.” are those with the dominance of human dimensions rather than traditional or pure biodiversity may be a reflection of this process.

The second most published topic “Urban & Spat.plan.” reflects the increasing recognition of the importance of spatial planning in influencing biodiversity and ecosystems services (Albert et al. 2020; Van der Biest et al. 2020). The topic “Economics & Cons.” is characterised by a high citation rate and this is the only topic with an increasing prevalence, reflecting that economic dimensions of conservation are crucial to its integration into policy priorities.

Climate change has been identified as a key pressure on biodiversity (summarised in Dìaz et al. 2019), and this is also reflected in policies (e.g. The European Green Deal EC 2019). There have been repeated calls to better integrate climate-related issues into science and policy (e.g. Pettorelli et al. 2021). The “Species & Climate change” topic gained the highest citations rates per article; twice as much as any of other topics, although the annual citations show a decreasing tendency. This may be a result of some articles with exceptionally high influences during the climate debate. The term “climate” appears among the top 100 common terms in five other topics as well. Our analysis thus provides some evidence of an integrated approach to climate change, biodiversity and ecosystems services, in particular the interactions between climate and species distribution.

Agriculture is one of the main pressures on ecosystem services and biodiversity (e.g. Diaz et al. 2019). Agriculture is also one of the main beneficiaries of nature’s contribution to people, and therefore sustainable production is one of the key issues and opportunities for biodiversity (IPBES 2018, 2019). This is reflected in major policies e.g. Common Agricultural Policy (2021–2027) and The European Green Deal (EC 2019). The topic “Agriculture” showed the highest prevalence among all topics (although with a decreasing relative frequency over time); moreover, this topic had a relatively high citation per article.

The shift towards a “natural capital” approach might be a reason why the second topic with conservation among the ten most common words, “Cons. & Distributions”, was a far less “hot topic” compared to “Economics & conservation” (although nature conservation-related terms appeared in four more topics among the top 100). “Cons. & Distributions” and “Diversity & Plants” are two topics that might be considered closer to the core of pure biodiversity research and had fewer human implications than other topics, and we conjecture that this may be a reason why these topics were less “hot”.

The topics “Carbon & Soil & Forestry” and “Hydro- & Microbiology” showed a relatively low performance according to our metrics (low number of articles, coupled with low prevalence although accompanied by a relatively high citation score). Despite the clear importance of soil biology in carbon capture, flood mitigation and agricultural productivity (Pereira et al. 2018), as well as the wider role of microbial diversity (Antwis et al. 2017), this finding suggests that soil is under-researched. Soil has been somewhat neglected in the biodiversity policy sphere: it is barely mentioned in the Aichi targets and has only begun to gain prominence within CBD reporting in the 2021 first draft (CBD 2010, 2021a,b). In contrast, we found “Hydro- and Microbiology” is slightly more cited, and citation increases with the number of publications. This topic is distinct from the previous topic, despite the overlap of some properties between soil and water such as soil sealing and runoff. Given the increased prevalence and intensity of flood events in Europe and North America, there may well be greater policy-led demand for research on the interface of soil biology, hydrology and hydrobiology. This has been represented in a recent debate over the concept of Nature-based Solutions; a potential win–win situation for biodiversity and flood management (Opperman et al. 2009; Turkelboom et al. 2021). Considering the fact that many of the current emerging topics in the field of global conservation issues, identified by Sutherland et al. (2021) fit topics “Hydro- and Microbiology”, one would expect a higher future performance of this topic, in comparison with others.

In addition to the topics that came out of our analysis, it is interesting to note some potential topics that did not. The lack of representation of genetic diversity among the top 10 terms is perhaps unsurprising given its relative neglect in policy frameworks such as the CBD (Hoban et al. 2020). Co-development and co-production were likewise absent: the need for more co-development in biodiversity research has been highlighted by other authors (Mupepele et al. 2021; O'Brien et al. 2021). More unexpected was the lack of the words ‘animal’ (lacking even from the top 100 terms) though this may be an artefact resulting from a tendency of zoologists to use finer taxonomic scales (e.g. bird, insect or bee, rather than animal). On the contrary, ‘plant’ appeared amongst the most common words in the topic “Diversity & Plants”. Although clearly a key part of biodiversity, plants are sometimes reported upon separately (e.g. Global Strategy for Plant Conservation, reviewed by Sharrock 2020) and this approach may in part account for the status of botany we observed. Among the top 100 terms, only some of the main taxonomic groups, mainly those with importance for Ecosystem Services as “invertebrate”, “bird”, “insects”, ” fish”, “reef”, “bee”, “flower” and “bacteria” appear, while other groups such as “fungi”, “mammals”, “amphibians” and “herpetofauna” are missing.

Within the ecosystem service categories mentioned in CICES 5.1., only “pest control”, “pollination”, “agricultural and forest and aquaculture production” and “nutrient cycling” appear among the top “terms”. Other categories such as “wind protection”, “nursery” or “cultural services” are not present among the most frequent words. This suggests something of a disconnect between the terminology of biodiversity and ecosystem services. The only topic where recognisable ecosystem service terms (CICES 5.1. categories) are completely missing among the top 100 terms is in the topic “Conservation and Distribution”.

It would be interesting to rerun these analyses in the future to see if such topics and others highlighted in horizon scanning exercises based on expert interviews using the Delphi method (e.g. Sutherland et al. 2021) emerge. Post hoc testing of horizon scanning using text mining would help to quantify its effectiveness, potentially identify biases and ultimately improve their predictive power. We note, however, that there are biases inherent in our approach, not least of which is its limitation to publications in English (Amano et al. 2021). While English may be the lingua franca of science, many policy-makers understandably prefer to read texts in their native language and thus the influence of papers in these languages may well be important.

## Conclusion

We have shown that text mining can provide insights into trends in research. When a research field includes tens of thousands of papers, applying automated analyses is an alternative of subjective shortening the document list. We applied this technique to the documents containing ecosystem service and biodiversity research. However, as there is no generally accepted protocol for text mining techniques yet, further research is needed in defining the least number of eligible topics (number of meaningful clusters), and also for a standardised bias testing between full-text and abstract-based analyses. As we had an ambitious goal reviewing a large amount of research, we faced difficulties in interpretation, which we tried to overcome with the help of a novel method. However, this would have been challenging without referring to our knowledge of events such as publication of key policy documents. Additionally, should the technique achieve wider use, a summarised indicator could be developed for comparison of “hot” and “cold” topics.

Our analysis found a marked increase in the number of publications bringing together biodiversity and ecosystem services. Out of the nine identified topics, “Science & Policy” is among the most numerous, best cited and the fastest growing topics, which might reflect demand from policy-makers and stakeholders for a rigorous evidence base. All other topics show differing performances in each of the indicators we used (number of articles, beta and gamma values, number of citations, temporal dynamics) and while it is not easy to give a comprehensive answer to the question of which topics are the “hot” and “cold” ones, it does offer insights into recent trends. “Hot topics” cover two concepts: frequency of papers which refer to that topic and the frequently with which they are cited. Both of these metrics offer insight into the research priorities of at the time. Number of articles may be the better reflection of research output but it is the number of citations that shows research influence.

The applied metrics showed a slightly better performance in the topics that had applied or human implications, compared to those topics with mainly pure scientific terms. Among the main ES production sectors, agriculture overwhelms the others (forestry and fishery). There were missing categories found in both taxonomic groups and ES categories.

As demonstrated in the case of biodiversity and ecosystems services, we believe text mining can suggest relationships between policy development and research agendas and perhaps crucially identify gaps where more knowledge may be needed to provide an evidence base. The technique, among others, can also be used to test horizon scanning and improve the quality of any literature review.

## Supplementary Information

Below is the link to the electronic supplementary material.

Supplementary file1 (XLSX 34 kb)

Supplementary file2 (PDF 1022 kb)
