# Supplementary material for: Trends and gaps in biodiversity and ecosystem services research: A text mining approach
Source: Ambio. 2022 Sep 3;52(1):81–94. doi: 10.1007/s13280-022-01776-2 (PMC9666618; doi:10.1007/s13280-022-01776-2)
Supplement: Supplementary file 2 — Supplementary file2 (PDF 1022 kb) [file 13280_2022_1776_MOESM2_ESM.pdf]

Trends and gaps in biodiversity and ecosystem services research: a text mining approach

Viktoria Takacs [1], C. David O'Brien [2,3]

1. Poznan University of Life Sciences, Faculty of Veterinarian and Animal Sciences, Department of Zoology, Wojska Polskiego 71/c Poznan 60625, Poland
2. NatureScot (Scottish Natural Heritage) Inverness, IV3 8NW, UK
3. Royal Botanic Garden Edinburgh, Edinburgh, EH3 5LR, UK

Acknowledgements

We would like to thank the editor and reviewers for their helpful comments. We are grateful to Piotr Cichocki, for introducing the methodology of topic modelling and to Danny O'Brien for helpful comments on the draft. V. Takacs partially was founded by project Ecoserv-Pol (Services provided by main types of ecosystems in Poland –an applied approach; Iceland Lichtenstein Norway grant ("Environment, Energy and Climate Change" EOG 2014-2021).

Supplementary Materials 2.

AMBIO

Trends and gaps in biodiversity and ecosystems services research: a text mining approach

Viktoria Takacs [1], C. David O'Brien [2,3]
1.Poznan University of Life Sciences, Faculty of Veterinarian and Animal Sciences, Department of Zoology, Wojska Polskiego 71/c Poznan 60625, Poland email: vikitakacs3@gmail.com
2. NatureScot (Scottish Natural Heritage) Inverness, IV3 8NW, UK
3. Royal Botanic Garden Edinburgh, Edinburgh, EH3 5LR, UK

Summary table of significant trends of Fig 3 and Fig 4.

**Variable**	**topic**	**F(1,19)**	**P**
The annual changes of topic prevalence Fig 3.	"Agriculture"	6.318	0.022
	"Species & Climate change"	8.007	0.012
	"Economics and conservation"	5.4	0.003
Number of citations (Fig 4/a)	"Cons. & Distribution",	5.032	0.038
	"Diversity & Plants",	7.249	0.014
	"Hydro- & Microbiology",	5.978	0.022
	"Research & Policy"	6.077	0.023
	"Urban & Spat. Plan"	6.980	0.017
Number of articles vs. citations Fig 4/b	"Diversity & Plants",	5.637	0.028
	"Research & Policy"	5.242	0.033
	"Hydro- & Microbiology"	5.138	0.035
